# Deeply sequenced metagenome and metatranscriptome of a biogas-producing microbial community from an agricultural production-scale biogas plant

**DOI:** 10.1186/s13742-015-0073-6

**Published:** 2015-07-30

**Authors:** Andreas Bremges, Irena Maus, Peter Belmann, Felix Eikmeyer, Anika Winkler, Andreas Albersmeier, Alfred Pühler, Andreas Schlüter, Alexander Sczyrba

**Affiliations:** 1 Center for Biotechnology, Bielefeld University, Bielefeld, 33615 Germany; 2 Faculty of Technology, Bielefeld University, Bielefeld, 33615 Germany

**Keywords:** Biogas, Anaerobic digestion, Wet fermentation, Methanogenesis, Metagenomics, Metatranscriptomics, Sequencing, Assembly

## Abstract

**Background:**

The production of biogas takes place under anaerobic conditions and involves microbial decomposition of organic matter. Most of the participating microbes are still unknown and non-cultivable. Accordingly, shotgun metagenome sequencing currently is the method of choice to obtain insights into community composition and the genetic repertoire.

**Findings:**

Here, we report on the deeply sequenced metagenome and metatranscriptome of a complex biogas-producing microbial community from an agricultural production-scale biogas plant. We assembled the metagenome and, as an example application, show that we reconstructed most genes involved in the methane metabolism, a key pathway involving methanogenesis performed by methanogenic *Archaea*. This result indicates that there is sufficient sequencing coverage for most downstream analyses.

**Conclusions:**

Sequenced at least one order of magnitude deeper than previous studies, our metagenome data will enable new insights into community composition and the genetic potential of important community members. Moreover, mapping of transcripts to reconstructed genome sequences will enable the identification of active metabolic pathways in target organisms.

## Data description

### Background

Production of biogas by anaerobic digestion of biomass is becoming increasingly important, as biogas is regarded a clean, renewable and environmentally compatible energy source [1]. Moreover, generation of energy from biogas relies on a balanced carbon dioxide cycle.

Biogas production takes place under anaerobic conditions and involves microbial decomposition of organic matter, yielding methane as the main final product of the fermentation process. Complex consortia of microorganisms are responsible for biomass decomposition and biogas production. The majority of the participating microbes are still unknown, as is their influence on reactor performance. Because most of the organisms in biogas communities are non-cultivable by today’s conventional microbiological techniques, sequencing of metagenomic total community DNA currently is the best way to obtain unbiased insights into community composition and the metabolic potential of key community members.

Here, we describe the deeply sequenced metagenome and metatranscriptome of an agricultural production-scale biogas plant on the Illumina platform [2]. We sequenced the metagenome 27X and 19X deeper, respectively, than previous studies applying 454 or SOLiD sequencing [3, 4], which focused primarily on community composition.

Metatranscriptomic sequencing of total community RNA, 230X deeper than previously reported [5], complements the metagenome. Combined, these data will enable a deeper exploration of the biogas-producing microbial community, with the objective of developing rational strategies for process optimization.

### Digester management and process characterization

The biogas plant, located in North Rhine Westphalia, Germany, features a mesophilic continuous wet fermentation technology characterized recently [6]. It was designed for a capacity of 537 *kW*_*e*_ combined heat and power (CHP) generation. The process comprises three digesters: a primary and secondary digester, where the main proportion of biogas is produced, and a storage tank, where the digestate is fermented thereafter.

The primary digester is fed hourly with a mixture of 72 % maize silage and 28 % liquid pig manure. The biogas and methane yields at the time of sampling were at 810.5 and 417.8 liters per kg organic dry matter (*l*/*kgoDM*), respectively. After a retention time of 55 days, the digestate is stored in the closed, non-heated final storage tank. Further information is summarized in Table 1.

**Table 1:** Characteristics of the studied biogas plant’s primary digester at the sampling date 15 November 2010

Process parameter	Sample
Net volume	2,041 *m* ^3^
Dimensions	6.4 *m* high, diameter of 21 *m*
Electrical capacity	537 *k* *W*_*e*_
pH	7.83
Temperature	40 °C
Conductivity	22.10 *m* *S*/*c* *m*
Volative organic acids (VOA)	5,327 *m* *g*/*l*
Total inorganic carbon (TIC)	14,397 *m* *g*/*l*
VOA/TIC	0.37
Ammoniacal nitrogen	2.93 *g*/*l*
Acetic acid	863 *m* *g*/*l*
Propionic acid	76 *m* *g*/*l*
Fed substrates	72 % maize silage, 28 % pig manure
Organic load	4.0 *k* *g* *o* *D* *M* *m* ^−3^ *d* ^−1^
Retention time	55 *d* *a* *y* *s*
Biogas yield	810.5 *l*/*k* *g* *o* *D* *M*
Methane yield	417.8 *l*/*k* *g* *o* *D* *M*

### Sampling and library construction

Samples from the primary digester of the biogas plant were taken in November 2010. Before the sampling process, approximately 15 *l* of the fermenter substrate were discarded before aliquots of 1 *l* were transferred into clean, gastight sampling vessels and transported directly to the laboratory.

For the metagenome, aliquots of 20 *g* of the fermentation sample were used for total community DNA preparation as described previously [7].

For the metatranscriptome, a random-primed cDNA library was prepared by an external vendor (Vertis Biotechnologie AG). Briefly, total RNA was first treated with 5^′^-P dependent Terminator exonuclease (Epicentre) to enrich for full-length mRNA carrying 5^′^ cap or triphosphate structures. Then, first-strand cDNA was synthesized using a N6 random primer and M-MLV-RNase H reverse transcriptase, and second-strand cDNA synthesis was performed according to the Gubler-Hoffman protocol [8].

### Metagenomic and metatranscriptomic sequencing

We sequenced one metatranscriptome and two metagenome shotgun libraries on Illumina’s Genome Analyzer IIx system, applying the Paired-End DNA Sample Preparation Kit (Illumina Inc.) as described by the manufacturer to generate 2×161 *bp* paired-end reads. On Illumina’s MiSeq system, we sequenced three further metagenome shotgun libraries, applying the Nextera DNA Sample Preparation Kit (Illumina Inc.) as described by the manufacturer to generate 2×155 *bp* paired-end reads. Our sequencing efforts, yielding 35 *Gbp* in total, are summarized in Table 2.

**Table 2:** Overview of the different sequencing libraries

Accession	Library name	Library type	Insert size ^∗^	Cycles	Reads	Bases
ERS697694	GAIIx, Lane 6	RNA, TruSeq	202±49	2×161	78,752,308	12,679,121,588
ERS697688	GAIIx, Lane 7	DNA, TruSeq	157±19	2×161	54,630,090	8,795,444,490
ERS697689	GAIIx, Lane 8	DNA, TruSeq	298±32	2×161	74,547,252	12,002,107,572
ERS697690	MiSeq, Run A1 ^*†*^	DNA, Nextera	173±53	2×155	4,915,698	761,933,190
ERS697691	MiSeq, Run A2 ^*†*^	DNA, Nextera ^*‡*^	522±88	2×155	1,927,244	298,722,820
ERS697692	MiSeq, Run B1 ^*†*^	DNA, Nextera	249±30	2×155	3,840,850	573,901,713
ERS697693	MiSeq, Run B2 ^*†*^	DNA, Nextera ^*‡*^	525±90	2×155	4,114,304	614,787,564

^*^Insert sizes determined with Picard tools. ^*†*^Partial runs. ^*‡*^This Nextera library was sequenced twice

### Metagenome assembly

Prior to assembly, we used Trimmomatic [9] version 0.33 for adapter removal and moderate quality trimming. After adapter clipping, using Trimmomatic’s *Truseq2-PE* and *Nextera-PE* templates, we removed leading and trailing ambiguous or low quality bases (below Phred quality scores of 3). Table 3 summarizes the effect on sequencing depth, more than 25 *Gbp* of sequence data passed quality control.

**Table 3:** Metagenomic and metatranscriptomic sequencing and quality control (QC)

Library type	Reads, raw	Reads, post-QC	Bases, raw	Bases, post-QC
Metagenome (total)	143,975,438	137,365,053	23,046,897,349	17,267,320,221
Metatranscriptome	78,752,308	73,165,986	12,679,121,588	8,455,809,264

We assembled the metagenome with Ray Meta [10] version 2.3.1, trying a range of *k*-mer sizes from 21 to 61 in steps of 10. To estimate the inclusivity of the set of assemblies, we aligned the post-quality-control sequencing reads to the assembled contigs with bowtie2 [11] version 2.2.4. We then used samtools [12] version 1.1 to convert SAM to BAM, sort the alignment file and calculate the mapping statistics. Given the total assembly size and contiguity and the percentage of mapped back metagenomic reads, we selected the assembly produced with a *k*-mer size of 31. Here, we assembled approximately 228 *Mbp* in 54,489 contigs greater than 1,000 *bp*, with an N50 value of 9,796 *bp*. 77 % (79 %) of metagenomic (metatranscriptomic) reads mapped back to this assembly.

### Gene prediction and annotation

We used MetaProdigal [13] version 2.6.1 to predict 250,596 protein-coding genes on the assembled contigs. We compared the protein sequences of all predicted genes with the KEGG database [14] release 72.0 using Protein-Protein BLAST [15] version 2.2.29+. Of the 250,596 predicted genes, 191,766 (76.5 *%*) had a match in the KEGG database using an E-value cutoff of 10^−6^. We determined the KEGG orthology (KO) for each gene by mapping the top-scoring BLAST hit to its orthologous gene in KEGG, resulting in 109,501 genes with an assigned KO. Table 4 summarizes our results.

**Table 4:** Metagenome assembly statistics, minimum contig size of 1,000 *b* *p*

Assembly metric	Our assembly
Total size	228,382,457 *b* *p*
Number of contigs	54,489
N50 value	9,796 *b* *p*
Largest contig	333,979 *b* *p*
Mapped DNA reads	105,461,596 (77 *%*)
Mapped RNA reads	57,436,058 (79 *%*)
Predicted genes	250,596
Of these, full-length	172,372 (69 *%*)
Match in KEGG Genes	191,766
Of these, assigned KO	109,501
Of these, in KEGG pathways	61,100

### Relating the metagenome and the metatranscriptome

**Fig. 1:**
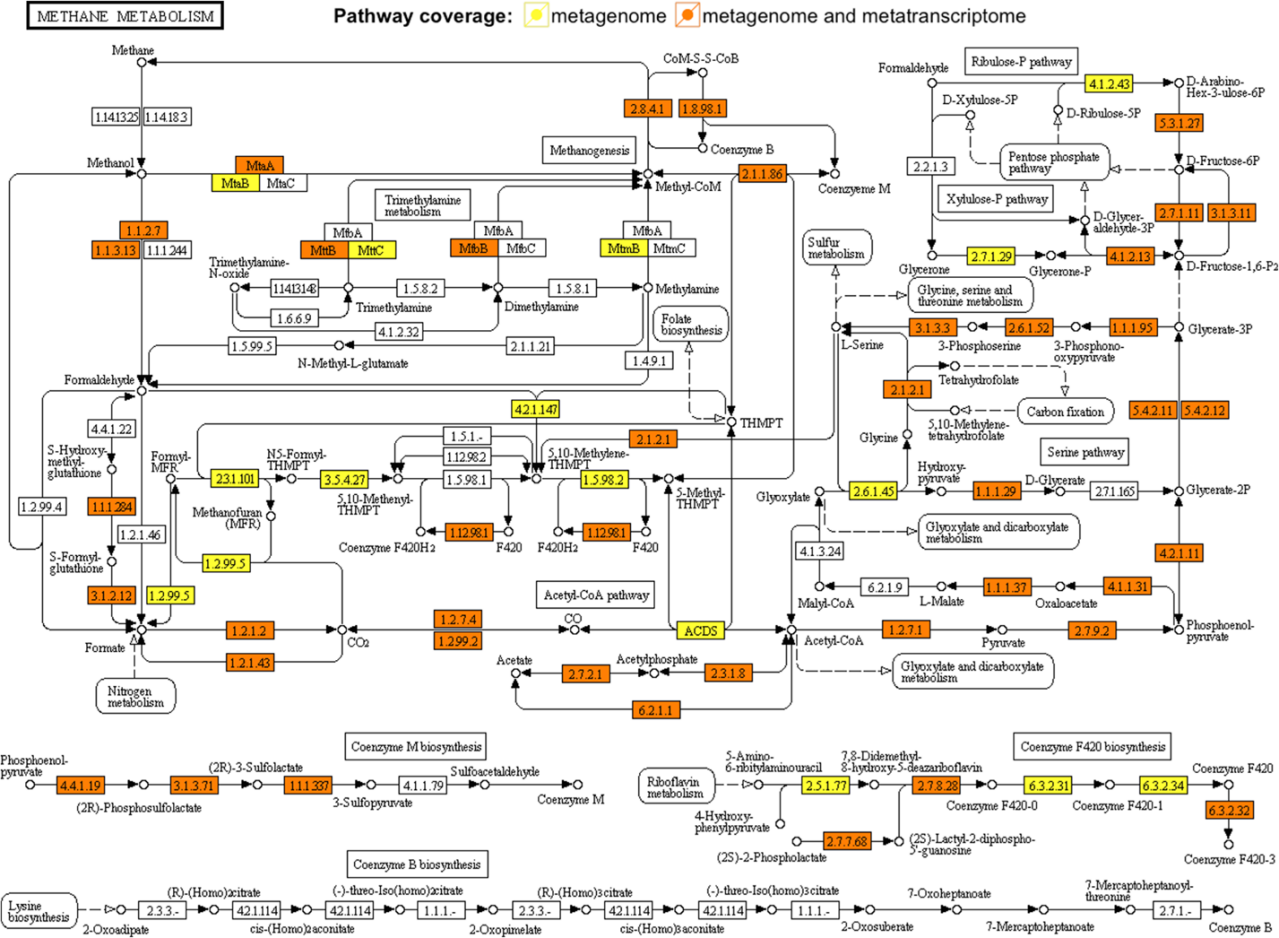
Methane metabolism pathway analysis. Genes reconstructed in our assembly that are involved in the methane metabolism [PATH:ko00680, (http://www.genome.jp/kegg-bin/show_pathway?ko00680)], are highlighted: genes with only metagenomic support are in yellow and genes with metatranscriptomic support as well, suggesting active gene expression, are in orange. Methane is synthesized from CO_2_, methanol or acetate. KEGG pathway map courtesy of Kanehisa Laboratories

To illustrate potential use cases, we first counted the number of reads within genes using BEDTools [16] version 2.22.0. The metagenomic and metatranscriptomic coverage of the methane metabolism pathway is shown in Fig. 1. This shows that we have assembled the majority of genes involved in the methane metabolism from our metagenomic data, with accompanying metatranscriptomic data suggesting active gene expression for many.

**Fig. 2:**
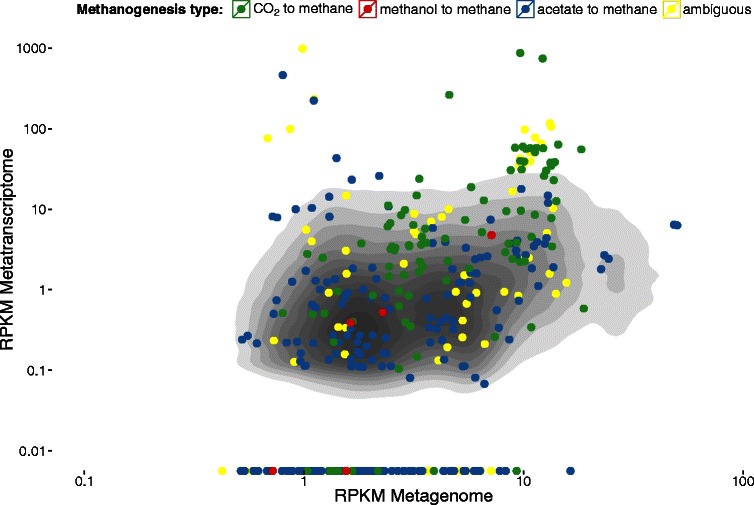
Relating the metagenome and metatranscriptome. Genes involved in methanogenesis are color coded by pathway type: CO_2_ to methane [MD:M00567, (http://www.kegg.jp/kegg-bin/show_module?M00567)] in green (96 genes), methanol to methane [MD:M00356, (http://www.kegg.jp/kegg-bin/show_module?M00356)] in red (5 genes) and acetate to methane [MD:M00357, (http://www.kegg.jp/kegg-bin/show_module?M00357)] in blue (209 genes). Common genes, shared between pathway types, are yellow (80 genes). In the background is a two-dimensional density estimation for all 250,596 genes

For a second example, we calculated the reads per kilobase per million mapped reads (RPKM) for each gene as a crude measure for abundance (metagenome) or expression (metatranscriptome). Figure 2 relates the two and highlights all genes assigned to any of the three known types of methanogenic pathways. Hydrogenotrophic methanogenesis, that is, the reduction of CO_2_ with hydrogen, appears to be highly expressed in the reactor analyzed, which is in agreement with results obtained by 454 amplicon and metatranscriptome sequencing [5].

## Discussion

We report extensive metagenomic and metatranscriptomic profiling of the microbial community from a production-scale biogas plant. Given the unprecedented sequencing depth and established bioinformatics, our data will be of great interest to the biogas research community in general and microbiologists working on biogas-producing microbial communities in particular. In a first applied study, our metagenome assembly was used to improve the characterization of a metaproteome generated from biogas plant fermentation samples and to investigate the metabolic activity of the microbial community [17].

By sharing our data, we want to actively encourage its reuse. This will hopefully result in novel biological and biotechnological insights, eventually enabling a more efficient biogas production.

## Availability of supporting data

### Data accession

Raw sequencing data are available in the European Nucleotide Archive (ENA) under study accession PRJEB8813 (http://www.ebi.ac.uk/ena/data/view/PRJEB8813). The datasets supporting the results of this article are available in *GigaScience*’s GigaDB [2].

### Reproducibility

The complete workflow is organized in a single GNU Makefile and available on GitHub [18]. All data and results can be reproduced by a simple invocation of *make*. To further support reproducibility, we bundled all tools and dependencies into one Docker container available on DockerHub [19]. *docker run* executes the aforementioned Makefile inside the container. Reproduction requires roughly 89 *GiB* memory and 83 *GiB* storage, and takes less than 24 hours on 32 CPU cores.

Excluding the KEGG analysis, which relies on a commercial license of the KEGG database, all steps are performed using free and open-source software.
